# Isotoxic stereotactic reirradiation for recurrent pelvic cancers^☆^

**DOI:** 10.1016/j.phro.2025.100889

**Published:** 2025-12-11

**Authors:** Christopher J.H. Pagett, John Lilley, Christopher O’Hara, Ane Appelt, Louise Murray, Rasmus Bokrantz, Jakob Ödén, Stina Svensson, Mark Harrison, Philip Camilleri, Rebecca Muirhead, Maxwell Robinson, Christopher Thompson

**Affiliations:** a Department of Medical Physics, Leeds Cancer Centre, Leeds Teaching Hospitals NHS Trust, Leeds, UK; b Leeds Institute of Medical Research at St James’s, University of Leeds, Leeds, UK; c Department of Clinical Oncology, Leeds Teaching Hospitals NHS Trust, Leeds, UK; d RaySearch Laboratories, Stockholm, Sweden; e Department of Oncology, Mount Vernon Centre for Cancer Treatment, Mount Vernon Hospital, Northwood, UK; f Department of Oncology, Churchill Hospital, Oxford University Hospitals NHS Trust, Oxford, UK; g Department of Oncology, University of Oxford, Oxford, UK; h Department of Radiotherapy Physics, Churchill Hospital, Oxford University Hospitals NHS Trust, Oxford, UK

**Keywords:** Isotoxic radiotherapy, Reirradiation, Recurrent pelvic cancer, Stereotactic body radiotherapy, Stereotactic ablative radiotherapy, Radiobiological optimisation, Support Tool for ReIrradiation Decisions guided by Radiobiology (STRIDeR)

## Abstract

**Background and purpose:**

Reirradiation is clinically challenging, requiring a balance between delivery of dose to tumour while respecting cumulative organ at risk (OAR) dose constraints. Standard prescriptions are often conservative, ignoring patient variability in achievable OAR doses. Isotoxic radiotherapy individualises treatment by delivering the highest equieffective dose in 2 Gy per fraction (EQD2Gy) while meeting OAR constraints. This technical feasibility study assessed isotoxic pelvic reirradiation using cumulative OAR constraints, the original dose distribution as background, and voxel-by-voxel EQD2Gy optimisation.

**Materials and methods:**

Data from 30 patients previously treated with pelvic stereotactic body radiotherapy (SBRT) at three UK centres were included. OARs were delineated on both previous and reirradiation image sets and deformably registered. Previous dose was mapped to the current image set and used as background dose for SBRT planning, following published methods. Initial 25 Gy in five fractions (25 Gy/5#) plans were generated for all patients, with further isotoxic dose escalation conducted up to a maximum of 50 Gy (fraction number fixed) until cumulative EQD2Gy constraints were reached.

**Results:**

For 25 of 30 patients, clinically acceptable isotoxic plans were obtained, with 23 exceeding the standard UK reirradiation prescription dose of 30 Gy/5#. The median isotoxic prescription was 42 Gy/5#, with four patient plans reaching the upper evaluated limit of 50 Gy. Vessels and the sacral plexus were most frequently dose limiting.

**Conclusion:**

This study highlighted the feasibility of isotoxic pelvic reirradiation and supports further investigation into automation and prediction models to streamline implementation in clinical practice.

## Highlights

• Radiobiological voxel-based planning used to guide isotoxic pelvic reirradiation.
• Dose from previous radiotherapy incorporated using deformable registration.
• Dose escalation feasible in 23/25 cases without exceeding organ at risk limits.
• It is feasible to safely personalise pelvic reirradiation dose between 30–50  Gy.
• Median escalated dose was 42  Gy; four patients reached the 50  Gy maximum dose.

## Graphical abstract

**Figure:**
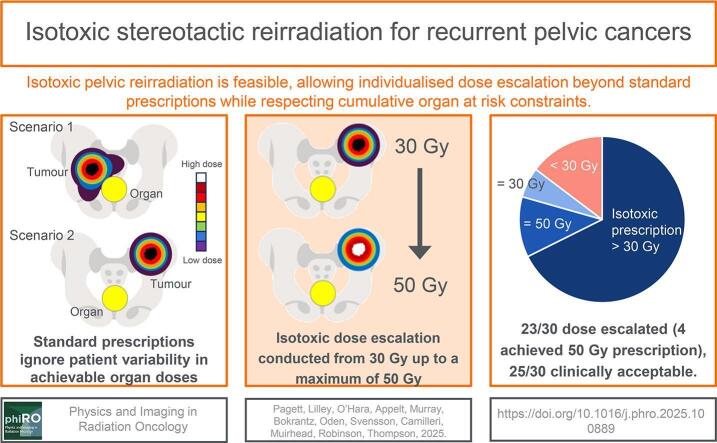

## 1 Introduction

Reirradiation, the delivery of further radiotherapy to an area previously irradiated, is an increasingly common scenario. A key challenge in reirradiation planning is meaningfully accounting for previous dose distributions within treatment plan optimisation. Previous organ at risk (OAR) dose limits how much can be safely delivered with reirradiation; but estimating locally delivered previous dose is often challenging. This problem is compounded by variations in tumour volume, location, dose prescription, and patient anatomy across both treatments. Consequently, reirradiation dose prescriptions are often conservative [1] and may not reflect the patient’s individual situation. While OAR constraints for pelvic reirradiation are poorly defined, cumulative constraints are often used, combining original and reirradiation doses (typically using equieffective dose in 2 Gy per fraction, EQD2Gy). This further complicates the planning process.

Isotoxic radiotherapy delivers the maximum tumour dose whilst meeting safety limits on OAR doses based on personalised evaluation of normal tissue dose, keeping it below predefined constraints [2]. The aim of isotoxic radiotherapy is to maximise local tumour control through individualised dose escalation. It is relevant for reirradiation, where standard dose prescriptions often fail to suit most patients’ OAR restrictions.

Isotoxic radiotherapy has proven feasible across many sites [3], [4], [5], [6], [7] and is under investigation in several trials [8], [9], [10], [11]. This evidence base supports isotoxic dose escalation as a promising strategy to safely increase tumoricidal dose and potentially improve outcomes. However, its use in reirradiation with cumulative OAR constraints remains very limited [12]. This is possibly because, until recently, it has been difficult to perform radiobiologically meaningful reirradiation planning efficiently, thus limiting isotoxic reirradiation implementation.

This technical feasibility study assessed isotoxic pelvic reirradiation using cumulative EQD2Gy OAR constraints by accounting for the delivered dose from the prior treatment as background dose and performing voxel-by-voxel EQD2Gy optimisation. It is based on a previously published research methodology (described below) [13] and evaluated on a multicentre patient cohort. In this context, our study advances isotoxic planning by applying cumulative, biologically informed optimisation to reirradiation, incorporating deformable dose summation, and demonstrating multicentre feasibility, thereby addressing some of the limitations in the existing literature.

## 2 Materials and methods

### 2.1 Patient cohort

Data from 30 previously reirradiated patients (planning CTs, plans, structures, and doses) were included from three UK centres: Leeds Teaching Hospitals (18), Oxford University Hospitals (7) and Mt Vernon Hospitals (5), Table S1. The Bradford Leeds Research Ethics Committee (#307580) approved the retrospective study. Clinically, all patients had limited volume oligometastatic or anastomotic pelvic recurrence after prior radical or adjuvant radiotherapy. All but one received 30 Gy in five fractions (30 Gy/5#) stereotactic body radiotherapy (SBRT) at reirradiation.

Patients had primarily been previously treated for prostate cancer with a prostate only field (n = 21) or a prostate and pelvic nodal field (n = 1) and the remainder treated for rectal cancer (n = 8). Most reirradiation targets represented isolated nodal recurrence (n = 24) with larger reirradiation targets in rectal cancer patients compared to prostate cancer. Overlap between primary and secondary planning target volumes (PTVs), when registered, ranged from 0-100 %. Most patients were treated supine for both courses, although some were treated prone for their primary (n = 5). Reirradiation was mostly delivered using a conventional linac (n = 25) with the remainder treated with CyberKnife. The entire cohort was categorised as type 1 reirradiation [14] as the main concern was overlap of irradiated volumes rather than volume effects in normal tissue (criteria for type 2). Full cohort characteristics are in Table S1.

### 2.2 Treatment planning and contouring

A research version of the RayStation (11A DTK; RaySearch, Stockholm, Sweden) treatment planning system was used to produce isotoxic plans via the STRIDeR (Support Tool for Reirradiation Decisions guided by Radiobiology) methodology [13]. This level of full integration is not currently available in other treatment planning systems. STRIDeR allows for the original dose distribution to be used as a background dose for reirradiation planning. Optimisation is performed voxel-by-voxel, with OAR optimisation objectives and functions evaluating cumulative dose in EQD2Gy [15]. STRIDeR allows the reirradiation dose prescription to be adjusted while keeping cumulative EQD2Gy OAR constraints constant, without extra radiobiological calculations.

A Medical Physicist reviewed all clinical OAR structures for accuracy, with missing structures contoured by an experienced consultant Clinical Oncologist. For this cohort, 27 of 30 patients had at least one overlap between reirradiation PTV and an OAR. Thirteen cases had two overlaps, six had three, and one had four. Of 47 overlaps, vessels (21) and sacral plexus (10) were most common, followed by colon (8), rectum (3), small bowel (3), bladder (1), and cauda equina (1). The PTV margin was 0.5 cm for all patients; however, 25 were expanded directly from the gross tumour volume (GTV) and five were expanded from the clinical target volume.

### 2.3 Image registration and dose constraints

The original CT was registered to the reirradiation CT using deformable image registration (DIR) in RayStation, following initial rigid image registration with the reirradiation CT as the reference image and the original CT as the target image, following a published method [16]. The DIR used ANACONDA/MORFEUS algorithms [17], [18] with registration quality checked by a Clinical Oncologist, and using mean distance to agreement (MDA) following a previously published approach [19]. See Supplementary Material for further details. Cumulative EQD2Gy D_0.1 cm3_ constraints for bladder, cauda equina, colon, rectum, sacral plexus, small bowel, vessels and D_10 cm3_ to femoral heads, plus α/β values, are provided in Table 1. EQD2Gy was calculated using the linear quadratic model. Constraints were obtained from de novo physical consensus [20] but relaxed per previous work [21]. Bladder was further relaxed based on consensus guidelines [22], vessels tightened based on thoracic reirradiation survey [23] and femoral heads adapted from a bone SBRT study [24]. Cauda equina and sacral plexus constraints incorporated 33 % recovery. DIR was unreliable for small bowel and colon dose estimation; for these, the original treatment maximum dose (D_0.1 cm3_) within 2 cm of the reirradiation PTV (mapped to the original scan) was used as background dose [13]. The selection of 2 cm was pragmatic, guided by typically rapid dose falloff of SBRT and investigated in previous work [25]. If no bowel was within this margin, background dose was presumed to be 0 Gy.

**Table 1:** Cumulative constraints used for the current study. All patients had an interval of at least 6 months from their original course of radiotherapy to reirradiation. Recovery was only applied to sacral plexus/cauda equina, and a total cumulative dose was used for other OARs. Constraints were obtained from de novo physical consensus but relaxed/tightened. Relaxed colon, rectum, small bowel, cauda equina and sacral plexus constraints were based on cautious interpretation/extrapolation from [[26], [27], [28], [29]]. Relaxed bladder was based on consensus guidelines [22] and relaxed femoral heads was adapted from a bone SBRT study [24]. Finally, tightened vessels were based on thoracic reirradiation survey [23].

OAR	Constraint (Gy)	Volume (cm^3^)	α/β value (Gy)	Constraint EQD2Gy (Gy)	Adjusted constraint EQD2Gy (Gy)
Bladder	38	D0.1	3	80.6	110.0
Cauda Equina	32	D0.1	2	67.2	67.2 with 33 % recovery
Colon	32	D0.1	3	60.2	89.9
Femoral Heads	30	D10	3	54.0	70.0
Rectum	32	D0.1	3	60.2	89.9
Sacral Plexus	32	D0.1	2	67.2	67.2 with 33 % recovery
Small Bowel	30	D0.1	3	54.0	78.8
Vessels	53	D0.1	3	144.2	110.0

### 2.4 Planning strategy and evaluation

SBRT planning was used, with centrally peaked dose and rapid falloff to cover the full PTV. An experienced Medical Physicist generated all plans. A 6MV flattening filter-free beam energy with a 0.2 cm dose grid resolution was used. Volumetric modulated arc therapy with sliding window sequencing was used, with either a 200° arc for lateral tumours or a 360° arc for visually-assessed central tumours.

For all plans, the target objectives were optimised in physical dose (considering only the reirradiation dose delivery) whereas the OAR objectives were optimised using cumulative EQD2Gy (reirradiation dose delivery combined with background prior radiotherapy given de novo) [15]. The target objectives were (as per local practice): >95 % of PTV should receive prescription dose, ≤0.5 cm^3^ of the GTV should receive 130 % of the prescription dose, and ≤ 0.1 cm^3^ of PTV-GTV should receive 130 % of the prescription dose.

Before isotoxic planning, a SBRT (25 Gy/5# [20]) plan was produced as a minimum dose plan. This was used to investigate if the case met the requirements for isotoxic dose escalation: (1) none of the cumulative OAR EQD2Gy D_0.1 cm3_/D_10 cm3_ constraints exceeded, (2) PTV D_95%_ > 25 Gy, (3) the 50 % isodose (50 % of prescription dose (Px)) contained within 2 cm margin from PTV, and (4) the maximum dose to the GTV and PTV-GTV less than 130 % of Px (to D_0.5 cm3_ and D_0.1 cm3_, respectively).

If the 25 Gy/5# plan failed to meet requirement (1) without breaching (2–4), the patient was deemed ineligible for reirradiation under this study’s criteria. For eligible cases, a dose-escalated isotoxic plan was attempted. Here, the prescription dose was increased in increments of 5 Gy until the above criteria could not be met, then by 1 Gy increments from the last successful 5 Gy threshold level, up to a maximum of 50 Gy. In all cases where dose escalation beyond 25 Gy was achievable, a 30 Gy/5# plan was also generated (reflecting a commonly adopted reirradiation dose prescription). Plans were evaluated using local clinical pre-treatment QA standards.

In total, 25 clinically acceptable plans were produced. For five cases it was not possible to achieve at least 95 % PTV coverage at 25 Gy without exceeding cumulative OAR constraints. These five cases were therefore excluded from the following results. For the remaining cases, it was possible to achieve at least 95 % PTV coverage at 30 Gy. Final isotoxic plans were visually evaluated by an experienced consultant Clinical Oncologist. Cumulative EQD2Gy dose distributions from the previous and isotoxic plans were generated for each patient, following methods previously described [16]. The D_0.1 cm3_/D_10 cm3_ was verified for each OAR to ensure that the value did not exceed the cumulative EQD2Gy dose constraint and so confirm that the research planning method was performing as expected.

### 2.5 Data analysis

For each 30 Gy/5# and final isotoxic plan, D_95%_ for the PTV and D_0.5 cm3_ for GTV were recorded alongside cumulative OAR EQD2Gy values. Dose metrics (D_0.1 cm3_, D_10 cm3_ and D_95%_) were compared between the clinically delivered 30 Gy/5# plans and the 30 Gy plans generated using STRIDeR EQD2Gy background dose optimisation, and the 30 Gy plans STRIDeR and isotoxic plans also produced with STRIDeR methodology (both produced using EQD2Gy background dose optimisation), using paired sample tests. Given the small sample size and the heterogeneity of the patient cohort, the data distributions were compared with the non-parametric Wilcoxon signed-rank test. Median of the differences and interquartile ranges were calculated. All statistics were performed in Visual Studio Code using Python statistics packages (SciPy.Stats).

## 3 Results

For most of the OARs, there were no significant differences between the clinically-delivered 30 Gy plans, and the 30 Gy plans generated using EQD2Gy background dose optimisation (p = 0.22–0.87 across bladder, colon, femoral heads, rectum, sacral plexus, small bowel). The cauda equina received a higher dose in the clinical plans (median 0.1 Gy vs. 0.0 Gy; p = 0.02), although absolute doses were low in both, and the differences were not clinically meaningful. Comparing target doses, the EQD2Gy plans delivered higher doses, with significant increases in GTV D_0.5 cm3_ (clinical 33.5 Gy vs. EQD2Gy 37.0 Gy; p < 0.01) and PTV D_95%_ (30.3 Gy vs. 32.0 Gy; p < 0.01). The detailed results are shown in Table 2.

**Table 2:** Planning results. Cumulative doses for OARs in EQD2Gy; and reirradiation plan GTV and PTV doses in physical dose.

Structure	Volume (cm^3^)	Clinically-delivered reRT median (Gy)	30 Gy plans generated using EQD2Gy background dose optimisation median (Gy)	Isotoxic median (Gy)	Clinically-delivered vs. 30 Gy plans generated using EQD2Gy background dose optimisation p-value	30 Gy plans generated using EQD2Gy background dose optimisation vs. Isotoxic p-value	30 Gy plans generated using EQD2Gy background dose optimisation vs. Isotoxic Median of differences (Gy) (Interquartile range)
Bladder	D_0.1_	6.2	5.7	8.0	0.51	<0.01	1.3 (0.2–4.3)
Cauda Equina	D_0.1_	0.1	0.0	0.0	0.02	0.07	0.0 (0.0–0.0)
Colon	D_0.1_	11.4	10.8	14.2	0.22	<0.01	2.7 (0.4–5.4)
Left Femoral Head	D_10_	0.4	0.6	0.8	0.55	<0.01	0.2 (0.0–0.6)
Right Femoral Head	D_10_	1.0	0.6	0.8	0.58	<0.01	0.2 (0.0–0.9)
Rectum	D_0.1_	6.8	6.5	8.9	0.33	<0.01	1.6 (0.8–3.4)
Sacral Plexus	D_0.1_	18.2	17.9	21.2	0.71	<0.01	3.8 (0.2–6.0)
Small Bowel	D_0.1_	8.2	7.8	9.0	0.87	<0.01	1.1 (0.4–3.4)
Vessels	D_0.1_	32.1	35.5	43.3	<0.01	<0.01	7.8 (4.1–9.1)
GTV	D_0.5_	33.5	37.0	47.0	<0.01	<0.01	10.0 (3.1–16.7)
PTV	D_95_ (%)	30.3	32.0	42.0	<0.01	<0.01	9.6 (4.4–11.9)

Of the 25 cases with 30 Gy plans generated for the current study, it was possible to isotoxically dose escalate in 23. Of those 23, it was possible to escalate to the maximum allowable prescription evaluated (50 Gy (10 Gy/#)) in four cases. The median isotoxic PTV D_95%_ was 42 Gy (8.4 Gy/#) vs. 32 Gy (6.4 Gy/#) for EQD2Gy 30 Gy plans (p < 0.01), with a median increase of 9.6 Gy (IQR 4.4–11.9). The isotoxic prescription for each patient is shown in Table S1 and the median isotoxic dose values are displayed in Table 2. The most common OARs to limit dose escalation were vessels (9), sacral plexus (9), small bowel (4), colon (2), bladder (1). Cauda equina and femoral heads did not limit escalation.

**Fig. 1:**
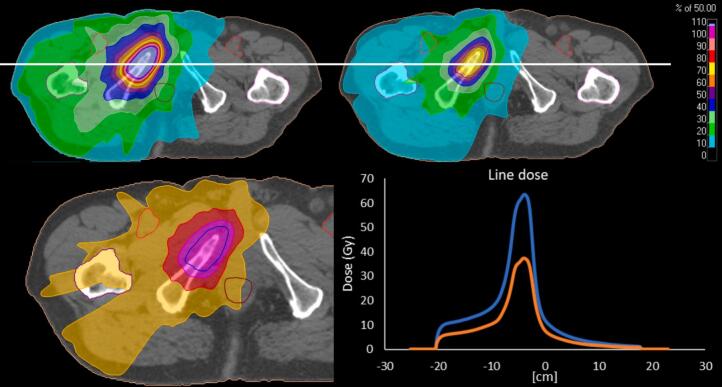
Dose distribution showing difference between isotoxic plan (50 Gy, top left) compared to 30 Gy plans generated using EQD2Gy background dose optimisation 30 Gy plan (top right). Also displayed is the absolute difference in dose (bottom left) and a transversal line dose (bottom right) showing the differences between 50 Gy (blue) and EQD2Gy background dose optimisation 30 Gy (orange) for the same patient. A white solid line indicates where the transversal line dose was recorded with 0 cm at the centre of the PTV. Structures included: PTV (dark blue), rectum (dark red), vessels (light red), left femoral head (pink), right femoral head (purple). (For interpretation of the references to colour in this figure legend, the reader is referred to the web version of this article.)

An example of dose distribution differences between a 30 Gy plan (generated for the current study) and 50 Gy isotoxic plan is shown in Fig. 1. OAR and target DVH metrics for 30 Gy plans generated using EQD2Gy background dose optimisation and isotoxic plans are compared in Table 2. There was a substantial difference between every OAR and GTV/PTV (p < 0.01), except for cauda equina (p = 0.07). Median increases were 1.3  Gy (IQR 0.2–4.3) for bladder, 2.7  Gy (0.4–5.4) for colon, 0.2  Gy (0.0–0.6) for left femoral head, 0.2  Gy (0.0–0.9) for right femoral head, 1.6  Gy (0.8–3.4) for rectum, 3.8  Gy (0.2–6.0) for sacral plexus, 1.1  Gy (0.4–3.4) for small bowel, and 7.8  Gy (4.1–9.1) for vessels. Target dose increases were 10.0  Gy (3.1–16.7) for GTV and 9.6 Gy (4.4–11.9) for PTV. However, despite the increase, evaluation of the cumulative original and isotoxic dose distributions demonstrated that cumulative OAR constraints were not exceeded in any cases.

All plans were deliverable, assessed using local clinical tolerances. All 25 isotoxically dose-escalated plans were deemed to have acceptable coverage when visually reviewed by a consultant Clinical Oncologist.

## 4 Discussion

This study demonstrates the feasibility of our EQD2Gy background dose optimisation methodology for isotoxic pelvic reirradiation SBRT planning across a heterogeneous cohort. Data from 30 reirradiation patients across three UK centres were used to generate isotoxic plans using voxel-by-voxel EQD2Gy optimisation with original doses as background. Dose escalation was achieved while maintaining cumulative EQD2Gy OAR constraints, with 23 of 25 cases exceeding a standard 30 Gy/5# prescription. The median isotoxic prescription reached 42 Gy, with four cases achieving the maximum tested 50 Gy. Our approach provided a systematic, efficient method for isotoxic reirradiation planning, eliminating the need for manual equieffective dose recalculations.

Isotoxic dose escalation ranged from EQD2_α/β = 10_ 40–83.3 Gy (EQD2_α/β = 1.5_ 64.3–164.3 Gy), with median of EQD2_α/β = 10_ 64.4 Gy (EQD2_α/β = 1.5_ 118.8 Gy). These doses are likely sufficient for medium- to long-term control depending on tumour histology. For recurrent prostate cancer, cohorts treated with EQD2_α/β = 1.5_ of 64.3–108.6 Gy achieved 3-year recurrence-free survival (RFS) of 79 % and overall survival (OS) of 87 % [[30], [31], [32]]. Locally recurrent rectal cancer treated to EQD2_α/β = 10_ of ∼ 40 Gy achieved 2-year OS of 73–77 % and local control (LC) of 71.6 % [[33], [34]]. Other pelvic, nodal, or bone recurrences, EQD2_α/β = 10_ has ranged from 37.3–60 Gy, with 2-year LC of 89–89.5 % and 1–2-year OS of 43–74.9 % [[35], [36], [37]]. Overall, the isotoxic EQD2Gy range achieved here generally exceeds or encompasses reported ranges, suggesting potential gains in LC and OS compared to EQD2_α/β = 10_ 30–40 Gy [[12], [38], [39]] particularly for rectal and other pelvic/nodal/bone recurrences.

Plans were developed using an SBRT technique, while preserving cumulative OAR constraints. Median PTV D_95%_ increased from 32  Gy to 42  Gy. Summed EQD2Gy distributions demonstrated cumulative OAR constraints were respected. As no broad international consensus exists for pelvic reirradiation constraints [[16], [40], [41]], conservative dose limits were used to evaluate the planning method rather than define optimal thresholds. Clinical practice varies across centres, including those contributing data. Our approach fixes cumulative OAR constraints while adjusting prescriptions, requiring changes only to PTV goals and improving planning efficiency compared with manual radiobiological correction, with further gains possible through automation [[42], [43], [44]]. Furthermore, maximum validated cumulative constraints are displayed after each optimisation, removing repeated cumulative dose generation.

Our cumulative dose constraints were adapted from de novo SBRT constraints [20] to allow a degree of dose escalation (except for vessels which was tightened). Without relaxation, the isotoxic prescription achievable would have been lower. Future work will be to compare these results with unrelaxed constraints. All reirradiation plans passed de novo limits. For this study, 50 Gy/5# was set as the upper limit for escalation, replicating previous work [12]. In four cases reaching this prescription, further escalation may have been possible with more lenient limits [[22], [26]]. Median PTV D_95%_ increased by 10 Gy (32 Gy to 42 Gy). Median OAR EQD2Gy differences were small, except vessels (7.7 Gy) and sacral plexus (3.9 Gy), which frequently overlapped the PTV and commonly limited escalation.

The cohort was heterogenous, with differences in reirradiation target location, patient orientation, and treatment technique. In five cases where 25 Gy/5# could not meet the study constraints, 30 Gy/5# were delivered clinically. Of these, three had substantial target overlap with the original PTV, Table S1, making the outcome unsurprising. For cases replanned to 30 Gy, OAR dose differences between clinical and EQD2Gy plans were minor. Cases were planned using an SBRT technique, both clinically and using our research methodology, with the research-planned GTVs and PTVs receiving higher doses, reflecting more appropriate application of OAR doses isotoxically using our approach. This also explains the increased vessel dose since these commonly overlapped with the GTV/PTV.

Acceptable OAR dose depends on expected clinical benefit. Where alternatives are limited or benefit is high, more lenient constraints may be justified, whereas stricter limits may be appropriate when benefit is uncertain. This highlights the need for individualised thresholds. While this study focussed on point-dose constraints, dose-volume constraints may better predict toxicity for organs, for example bladder, rectum, and bowel [[45], [46]]. A brief investigation of dose-volume constraints is included in the Supplementary Material.

Isotoxic rectal cancer reirradiation has previously been explored [12], assuming 15 % annual OAR repair compared to our work, which adopted a different approach. The authors also required mandatory PTV coverage of 60 % receiving prescription dose (compared to 95 % in this current study) and found the median EQD2_α/β = 10_ delivered to 80 % of the PTV increased from 43 Gy to 61 Gy. Performing a similar calculation for this study (i.e. calculating EQD2_α/β = 10_), median EQD2Gy to 95 % increased from 43.7 Gy to 64.4 Gy, which is comparable. This current work expands on prior work through multi-centre data and principally using voxel-by-voxel EQD2Gy optimisation. Dose escalation to 50 Gy/5# is more relevant in the context of large-volume or radioresistant recurrences, such as in rectal cancer. In prostate nodal recurrences, where the disease burden is often lower and radiosensitivity higher, 30 Gy/5# may be sufficient.

Accurate dose mapping from prior treatment is critical to respect cumulative OAR constraints. DIRs were visually assessed and quantitatively evaluated using MDA [[47], [48]], Supplementary Material. Given DIR uncertainties, especially for mobile organs, future work will incorporate robustness into the planning tool.

Limitations include uncertainties in reirradiation constraints and centre-specific protocols. Alternative isotoxic strategies, such as fractionation changes or constraint adjustment, should be explored. International consensus on validated constraints, outcomes, and tissue recovery modelling is needed, and this work may help inform future prospective trials. In situations where 95 % coverage was not possible, prescriptions were de-escalated instead of testing target coverage compromise, which warrants further investigation.

In conclusion, EQD2Gy background dose optimisation is feasible for isotoxic pelvic reirradiation SBRT. Dose escalation beyond 30 Gy/5# was achieved in 23/25 cases, with median doses reaching 42 Gy., while maintaining cumulative OAR constraints across centres. Automating voxel-level EQD2Gy optimisation improved planning efficiency and consistency. Future studies should validate clinical benefits and refine reirradiation constraints.

## CRediT authorship contribution statement

**Christopher J.H. Pagett:** Conceptualization, Methodology, Validation, Formal analysis, Investigation, Data curation, Writing – original draft, Visualization. **John Lilley:** Conceptualization, Methodology, Resources, Writing – review & editing, Supervision, Project administration. **Christopher O’Hara:** Investigation, Writing – review & editing. **Ane Appelt:** Conceptualization, Methodology, Writing – review & editing, Supervision, Funding acquisition. **Louise Murray:** Conceptualization, Methodology, Writing – review & editing, Supervision, Funding acquisition. **Rasmus Bokrantz:** Software, Writing – review & editing. **Jakob Ödén:** Software, Writing – review & editing. **Stina Svensson:** Software, Writing – review & editing. **Mark Harrison:** Writing – review & editing. **Philip Camilleri:** Writing – review & editing. **Rebecca Muirhead:** Writing – review & editing. **Maxwell Robinson:** Writing – review & editing. **Christopher Thompson:** Conceptualization, Methodology, Validation, Formal analysis, Investigation, Data curation, Writing – review & editing.

## Declaration of competing interest

The authors declare that they have no known competing financial interests or personal relationships that could have appeared to influence the work reported in this paper.
