# Supplementary material for: Isotoxic stereotactic reirradiation for recurrent pelvic cancers
Source: Phys Imaging Radiat Oncol. 2025 Dec 11;37:100889. doi: 10.1016/j.phro.2025.100889 (PMC12775924; doi:10.1016/j.phro.2025.100889)
Supplement: Supplementary Data 1 [file mmc1.docx]

Supplementary Material

Table S1: Patient cohort characteristics. *prostate and whole pelvis.

| Case | Original Orientation | Original Prescription (Gy/#) | Original PTV volume (cm^3^) | Reirradiation Site | Reirradiation Orientation | Reirradiation Modality | Reirradiation Prescription (Gy/#) | No. of reirradiation PTVs | Reirradiation PTV volume (cm^3^) | Overlap between original and reirradiation PTV as a % of reirradiation PTV volume | Distance between original and reirradiation PTV (cm) | Isotoxic  Prescription (Gy) |
| --- | --- | --- | --- | --- | --- | --- | --- | --- | --- | --- | --- | --- |
| Prostate | | | | | | | | | | | | |
| 1 | Supine | 37.5/15 | 261.4 | Node | Supine | Linac | 30/5 | 1 | 10.5 | 0.0 | 1.4 | 40 |
| 2 | Supine | 52.5/20 | 258.2 | Node | Supine | Linac | 30/5 | 1 | 7.1 | 0.0 | 0.4 | 46 |
| 3 | Supine | 52.5/20 | 264.0 | Bone (sacrum) | Supine | Linac | 25/5 | 1 | 57.5 | 0.0 | 2.4 | 31 |
| 4 | Supine | 52.5/20 | 310.8 | Node | Supine | Linac | 30/5 | 1 | 3.7 | 0.0 | 2.6 | 42 |
| 5 | Supine | 52.5/20 | 281.5 | Bone | Supine | Linac | 30/5 | 1 | 28.8 | 7.0 | 0.0 | 50 |
| 6 | Supine | 52.5/20 | 329.8 | Node | Supine | Linac | 30/5 | 1 | 14.1 | 0.0 | 4.3 | 32 |
| 7 | Supine | 55/20 | 293.1 | Bone | Supine | Linac | 30/5 | 1 | 18.4 | 0.0 | 2.7 | 50 |
| 8 | Supine | 55/20 | 233.6 | Node | Supine | Linac | 30/5 | 1 | 82.8 | 0.0 | 0.0 | 40 |
| 9 | Supine | 55/20 | 347.7 | Node | Supine | Linac | 30/5 | 1 | 13.0 | 0.0 | 2.4 | 36 |
| 10 | Supine | 55/20 | 262.2 | Node | Supine | Linac | 30/5 | 1 | 13.8 | 0.7 | 0.0 | - |
| 11 | Supine | 55/20 | 423.2 | Node | Supine | Linac | 30/5 | 1 | 8.2 | 2.6 | 0.0 | 44 |
| 12 | Supine | 60/20 | 209.7 | Node | Supine | Linac | 30/5 | 1 | 12.1 | 0.0 | 1.2 | 44 |
| 13 | Supine | 60/20 | 179.6 | Node | Supine | Linac | 30/5 | 2 | 10.0, 5.4 | 0.0 | 0.5 | 31 |
| 14 | Supine | 66/33 | 329.0 | Node | Supine | Linac | 30/5 | 1 | 11.6 | 3.6 | 0.0 | 42 |
| 15 | Supine | 66/33 | 336.2 | Node | Supine | Linac | 30/5 | 1 | 6.4 | 0.0 | 1.3 | 32 |
| 16 | Supine | 66/33 | 205.5 | Node | Supine | Linac | 30/5 | 2 | 5.8, 2.7 | 0.0 | 1.2 | 44 |
| 17* | Supine | 66/33 | 871.0 | Node | Supine | Linac | 30/5 | 1 | 8.2 | 0.0 | 0.4 | 41 |
| 18 | Supine | 66/33 | 267.1 | Node | Supine | Linac | 30/5 | 1 | 7.9 | 0.0 | 0.4 | 50 |
| 19 | Supine | 74/37 | 77.9 | Node | Supine | Linac | 30/5 | 1 | 19.6 | 0.0 | 1.5 | 37 |
| 20 | Supine | 74/37 | 180.2 | Node | Supine | Linac | 30/5 | 1 | 6.4 | 0.0 | 3.5 | 43 |
| 21 | Supine | 74/37 | 156.1 | Node | Supine | Linac | 30/5 | 1 | 20.2 | 0.0 | 4.0 | 43 |
| 22 | Supine | 74/37 | 131.4 | Bone | Supine | Linac | 30/5 | 2 | 17.5, 4.5 | 0.0 | 0.4 | 42 |
| Rectal | | | | | | | | | | | | |
| 23 | Prone | 25/5 | 1643.0 | Node | Supine | Linac | 30/5 | 1 | 12.3 | 0.0 | 0.2 | 40 |
| 24 | Prone | 25/5 | 1028.2 | Node | Supine | CyberKnife | 30/5 | 1 | 67.8 | 99.9 | 0.0 | - |
| 25 | Prone | 45/25 | 2012.9 | Node | Supine | Linac | 30/5 | 1 | 38.4 | 98.9 | 0.0 | 50 |
| 26 | Supine | 45/25 | 376.5 | Node | Supine | CyberKnife | 30/5 | 1 | 91.1 | 99.0 | 0.0 | - |
| 27 | Prone | 45/25 | 624.8 | Bone (sacrum) | Supine | CyberKnife | 30/5 | 1 | 138.4 | 0.0 | 0.2 | 30 |
| 28 | Supine | 45/25 | 2183.0 | Node | Supine | CyberKnife | 30/5 | 2 | 18.4, 24.7 | 2.5 | 0.0 | - |
| 29 | Prone | 50.4/28 | 1051.3 | Anastomotic recurrence | Supine | CyberKnife | 30/5 | 1 | 148.6 | 99.2 | 0.0 | - |
| 30 | Supine | 54/30 | 1039.1 | Node | Supine | Linac | 30/5 | 1 | 87.2 | 98.9 | 0.0 | 30 |

Description of contouring and image registration QA

The clinical team carefully reviewed OAR structures to ensure they were accurately and completely contoured. Edits were made as needed for consistency. Structures such as the rectum, bladder, small bowel (within the reirradiation target area), femoral heads, colon, and descending colon were delineated on both the original and reirradiation CT scans. Vessels, cauda equina, and sacral plexus were delineated only on the reirradiation scans.

To align the original CT with the reirradiation CT, rigid registration followed by deformable mapping was performed using the STRIDeR registration method, which accounts for bladder volume differences and anatomical changes due to surgery or organ movement. Key anatomical regions, including bones and parts of the bowel and bladder, were used to guide accurate registration. Medical physicists then visually inspected the results to ensure realistic anatomical alignment. MDA was used to quantitatively evaluate the deformable registrations, table S2. DIR was unreliable for small bowel and colon dose estimation; for these, the original treatment maximum dose (D0.1 cm^3^) within 2 cm of the reirradiation PTV (mapped to the original scan) was used as background dose. This approach removes the uncertainty of the deformable image registration providing a fixed background dose, therefore reducing the uncertainty in the summed dose; however, it is much more conservative than the DIR method and so may restrict dose escalation unnecessarily.

Table S2: Average MDA for OARs across patient cohort.

| Organ | Average MDA (mm) |
| --- | --- |
| Bladder | 1.7 |
| Cauda equina | 1.8 |
| Rectum | 1.5 |
| Sacral plexus | 1.9 |
| Vessels | 1.7 |

Investigation of volume constraints

Table S3: Assessment of cumulative dose-volume constraints (EQD2, α/β = 3 Gy) for bladder and rectum in selected cases using D30% thresholds from previous study [1].

| Case | Isotoxic Prescription (Gy) | OAR | Background D30% (Gy) | Cumulative D30% (Gy) | Constraint breached? | Notes |
| --- | --- | --- | --- | --- | --- | --- |
| 7 | 50 | Bladder | 38.0 | 38.2 | No | OARs not overlapping PTV; constraint not limiting |
|  |  | Rectum | 40.4 | 40.8 | No |  |
| 11 | 44 | Bladder | 62.3 | 64.1 | Yes | Constraint exceeded; would limit dose escalation |
| 15 | 43 | Bladder | 34.0 | 35.2 | No | Overlapping bladder, but constraint not exceeded |
| 18 | 50 | Bladder | 65.9 | 66.2 | Yes | Constraint (D30% < 57.9 Gy) exceeded prior to reirradiation |
|  |  | Rectum | 63.5 | 63.8 | No | Within constraint (D30% < 66 Gy) |
| 27 | 30 | Rectum | 43.8 | 45.8 | No | Overlapping rectum, but plan limited by another OAR (sacral plexus) |

Although this study evaluated only maximum point dose constraints, larger volume-dose constraints can influence isotoxic planning, especially in reirradiation scenarios. To explore this, some representative cases were reassessed using D30% constraints for bladder and rectum taken from a study on safe dose-volume limits for SBRT reirradiation [1]. The cumulative constraints (in EQD2, α/β = 3 Gy) were D30% < 57.9 Gy for bladder and D30% < 66.0 Gy for rectum. Other rectal dose-volume limits were provided in the source, but only D30% was considered for this limited analysis. The results are summarised in Table S3.

In all cases, the reirradiation plan contributed minimally to the cumulative OAR dose. The only two instances where the bladder D30% constraint was breached (cases 11 and 18) were due to high background doses; reirradiation itself remained within limits. These cases illustrate where isotoxic dose escalation would be restricted by prior dose exposure. In two cases (15 and 27), where bladder or rectum overlapped the reirradiation PTV, constraints were not breached. In case 27, the rectum D30% increased by 2 Gy, suggesting that volume-based constraints may impact isotoxic prescription in borderline cases. However, in this example, another OAR (sacral plexus) was the limiting factor.

[1] Augugliaro M, Marvaso G, Cambria R, Pepa M, Bagnardi V, Frassoni S, et al. Finding safe dose-volume constraints for re-irradiation with SBRT of patients with prostate cancer relapse: The IEO experience. Phys Med 2021;92:62–8. https://doi.org/10.1016/j.ejmp.2021.11.005.
